# Rare case report of main pulmonary artery occupying lesion: pulmonary artery intimal sarcoma

**DOI:** 10.1093/ehjcr/ytaf488

**Published:** 2025-09-30

**Authors:** Lei Liu, Ling Chen, Hui Chen, Jing Yao, Aijuan Fang

**Affiliations:** Department of Ultrasound Medicine, Nanjing University Medical School Affiliated Nanjing Drum Tower Hospital, No. 321 Zhongshan Road, Nanjing, Jiangsu Province 210000, China; Medical Image Center, Nanjing University Medical School Affiliated Nanjing Drum Tower Hospital, Nanjing, Jiangsu Province 210000, China; Department of Pathology, Nanjing University Medical School Affiliated Nanjing Drum Tower Hospital, Nanjing, Jiangsu Province 210000, China; Department of Ultrasound Medicine, Nanjing University Medical School Affiliated Nanjing Drum Tower Hospital, No. 321 Zhongshan Road, Nanjing, Jiangsu Province 210000, China; Medical Image Center, Nanjing University Medical School Affiliated Nanjing Drum Tower Hospital, Nanjing, Jiangsu Province 210000, China; Department of Ultrasound Medicine, Nanjing University Medical School Affiliated Nanjing Drum Tower Hospital, No. 321 Zhongshan Road, Nanjing, Jiangsu Province 210000, China; Medical Image Center, Nanjing University Medical School Affiliated Nanjing Drum Tower Hospital, Nanjing, Jiangsu Province 210000, China; Department of Ultrasound Medicine, Nanjing University Medical School Affiliated Nanjing Drum Tower Hospital, No. 321 Zhongshan Road, Nanjing, Jiangsu Province 210000, China; Medical Image Center, Nanjing University Medical School Affiliated Nanjing Drum Tower Hospital, Nanjing, Jiangsu Province 210000, China

**Keywords:** Pulmonary artery intimal sarcoma, Echocardiography, Pulmonary embolism, Tumor, Case report

## Abstract

**Background:**

Pulmonary artery intimal sarcoma (PAIS) is a rare aggressive malignant tumour that is easily confused with pulmonary embolism and poses considerable diagnostic challenges.

**Case summary:**

This case report details a 68-year-old male with progressive dyspnoea and chest tightness, initially misdiagnosed as pneumonia and pulmonary thromboembolism. Multimodal imaging, including echocardiography and contrast-enhanced computed tomography, identified an irregular mass within the pulmonary artery causing luminal stenosis, necessitating urgent surgical intervention. Histopathological examination confirmed PAIS. Postoperative recovery without adjuvant chemotherapy resulted in no recurrence or metastasis at a 2-year follow-up.

**Discussion:**

This case underscores the pivotal role of imaging in differentiating PAIS from thromboembolic conditions and highlights surgical resection as the cornerstone of treatment. Although the prognosis of PAIS remains poor, complete surgical excision may extend survival, emphasizing the importance of early multidisciplinary collaboration and long-term monitoring. This report advocates for increased clinical suspicion of PAIS in cases of refractory dyspnoea to reduce diagnostic delays.

Learning points

- Pulmonary artery intimal sarcoma (PAIS) should be considered in patients with unexplained dyspnoea and pulmonary artery obstruction, as early diagnosis and surgical resection are critical for improving outcomes.
- Multimodal imaging plays a pivotal role in distinguishing PAIS from thromboembolic conditions, emphasizing the need for heightened clinical suspicion in refractory cases.
- Early recognition of PAIS is crucial to avoid misdiagnosis and delays in treatment. Complete surgical resection remains the cornerstone for managing PAIS and improving survival.

## Introduction

Pulmonary artery intimal sarcoma (PAIS) is a rare and highly aggressive malignancy originating from the intimal layer of elastic arteries, most commonly the pulmonary arteries and aorta. Due to its nonspecific clinical presentation, PAIS is often misdiagnosed as more common conditions such as pulmonary embolism (PE), leading to delayed treatment and poor outcomes.^1–3^ This case report describes a 68-year-old male with PAIS, emphasizing the diagnostic challenges, the role of multimodal imaging, and the importance of surgical intervention. Additionally, this report discusses the broader implications of PAIS diagnosis and management, providing insights into differential diagnosis, multidisciplinary care, and the need for long-term follow-up.

## Summary figure

**Figure:**
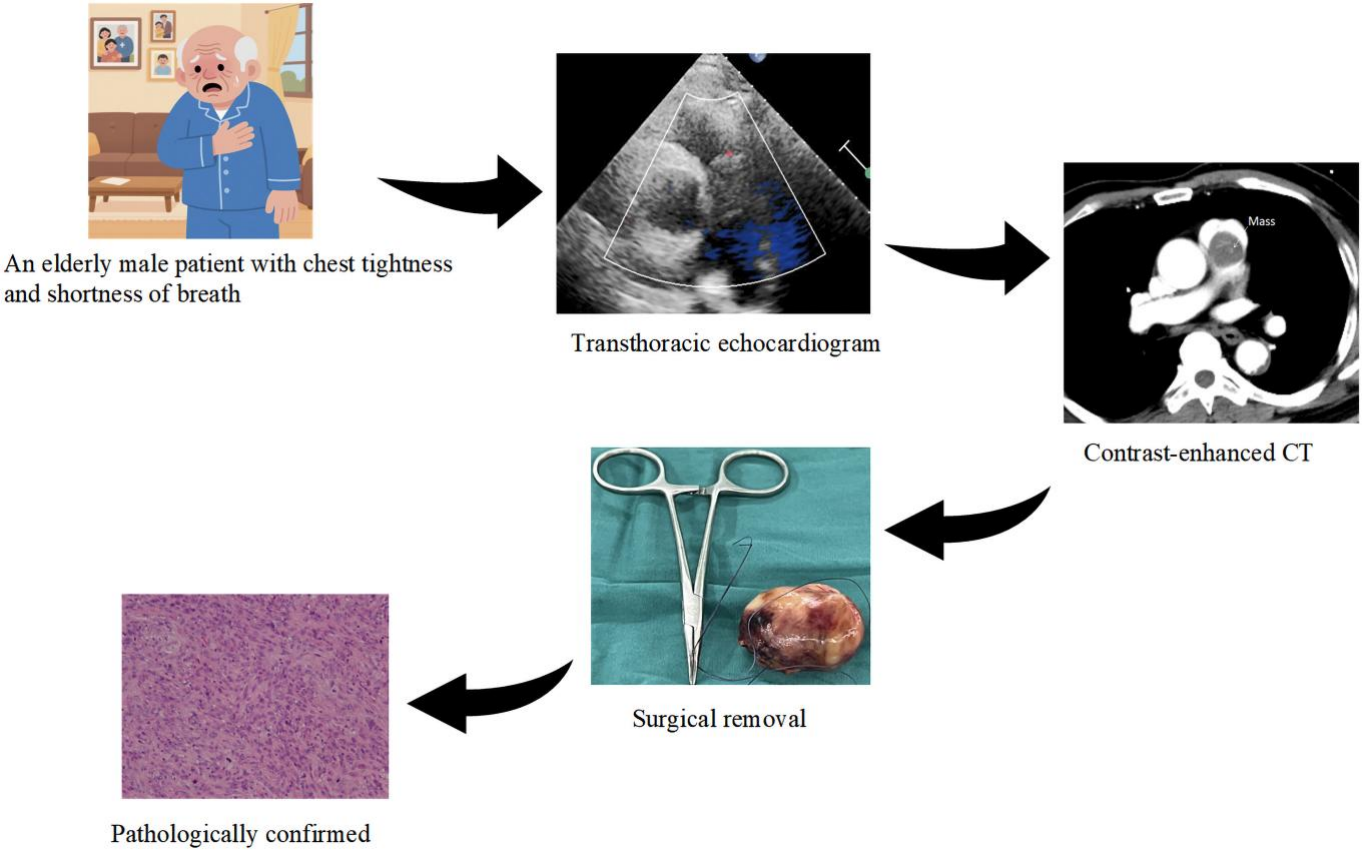


## History of presentation

A 68-year-old male presented with progressive dyspnoea, chest tightness, and shortness of breath over the past year, worsening with physical activity. He was initially diagnosed with pneumonia and pulmonary thromboembolism at a local hospital. Despite anti-infection and anticoagulation therapy, his symptoms continued to deteriorate over the next 2 months. He was subsequently referred to our hospital for further evaluation and management by the cardiac surgery department. On admission, physical examination revealed a pulse rate of 127 beats/min, a respiratory rate of 24 breaths/min, and a blood pressure of 132/74 mmHg (1 mmHg is ∼0.133 kPa). Systolic and diastolic murmurs were audible in the second intercostal space along the left sternal border.

### Past medical history

The patient was previously healthy with no reported medical history.

### Differential diagnosis

Auscultation revealed a significant cardiac murmur. Given the patient’s marked symptoms of chest tightness and shortness of breath, PE and valvular heart disease should be prioritized in the differential diagnosis.

### Investigations

**Figure 1:**
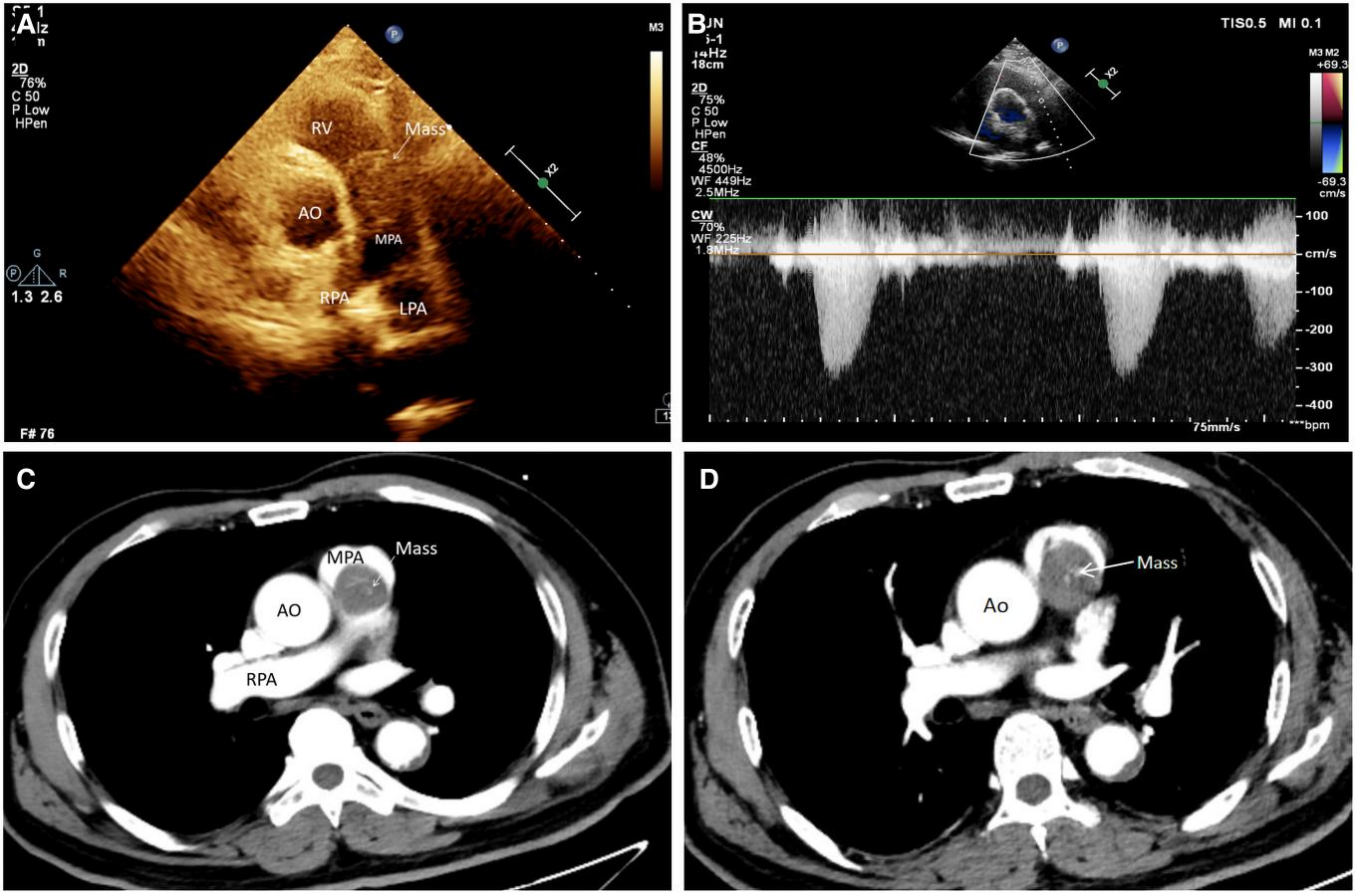
Multimodal imaging. (*A*) Transthoracic echocardiography shows a round-like mass located in the main pulmonary artery trunk. (*B*) Continuous Doppler shows increased blood flow at the mass. (*C* and *D*) Contrast-enhanced computed tomography of the chest shows a mass in the main pulmonary artery at two separate levels. Ao, aorta; RV, right ventricle; MPA, main pulmonary artery; LPA, left pulmonary artery; RPA, right pulmonary artery.

Transthoracic echocardiography (TTE) showed a widened pulmonary artery diameter (3.0 cm) and detected a moderately isoechoic, irregular mass measuring ∼3.38 × 2.39 cm in the main pulmonary artery near the pulmonary valve annulus (*Figure 1A*; *Videos 1* and *2*). The mass exhibited poor mobility, caused significant luminal stenosis of the proximal pulmonary artery trunk, and restricted pulmonary valve opening. Colour Doppler imaging revealed multicoloured mosaic flow signals near the mass, while continuous Doppler imaging showed an antegrade flow velocity of 3.3 m/s and a pressure gradient of 44 mmHg (*Figure 1B*). Electrocardiography indicated frequent ventricular premature contractions. Contrast-enhanced computed tomography (CT) of the chest identified a slightly hypointense, round lesion in the pulmonary valve area, with a filling defect visible on enhancement scans. The lesion was heterogeneous and slightly enhanced, measuring ∼29 × 30 mm (*Figure 1C* and *D*). A strip-like filling defect was also observed at the bifurcation of the main pulmonary artery and in both pulmonary artery branches. Based on these findings, clinicians initially suspected pulmonary artery tumour, and urgent surgical intervention was planned.

### Management

**Figure 2:**
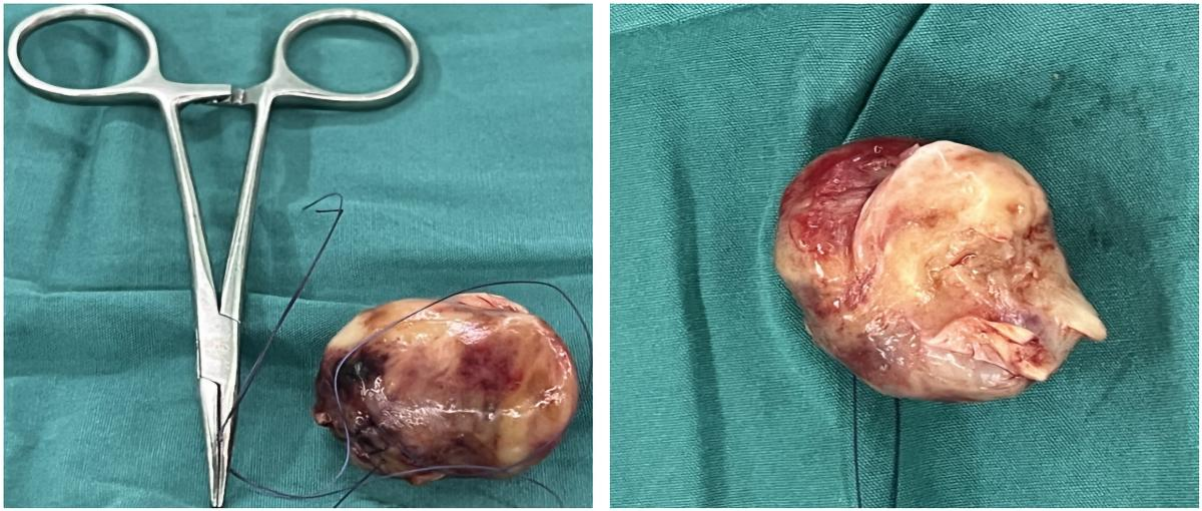
Solid specimens of pulmonary artery intimal sarcoma.

**Figure 3:**
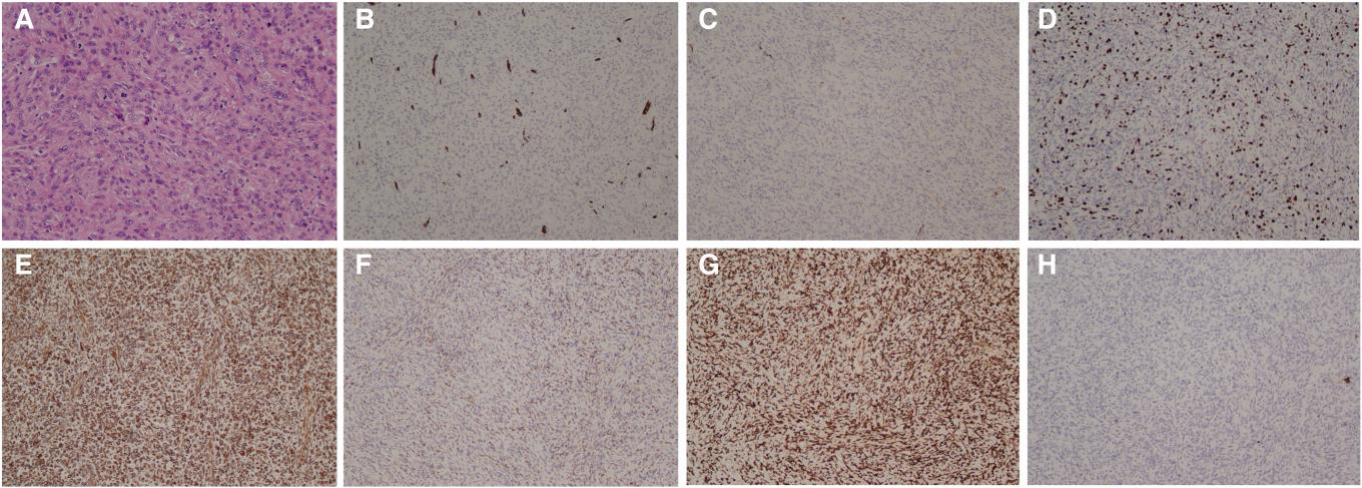
Pathological manifestations of pulmonary artery intimal sarcoma. (*A*) The PAIS is composed of spindle and pleomorphic cells with obvious atypia, ×200. (*B–H*) Microscopic results (×100) for CD34 (vascular+), S100 (scattered few+), Ki67 (30%+), vimentin (+++), CD68 (histiocyte+), CD163 (+++), and CK (−) in that order. PAIS, pulmonary artery intimal sarcoma.

During surgery, a brittle-textured mass measuring 6 × 5 cm with an enveloping outer surface was found in the main pulmonary artery trunk. Local endothelial hyperplasia was also noted. The tumour was completely excised, and the intima of the main pulmonary artery was stripped (*Figure 2*). Postoperative pathology revealed areas of cellular abundance interspersed with regions of sparse cellularity and interstitial collagenization. The tumour exhibited pleomorphic and heterogeneous cells, including spindle-shaped, ovoid, and polygonal forms, with visible nucleoli and frequent pathological mitoses (*Figure 3A*). Immunohistochemical staining showed that the tumour cells were negative for smooth muscle actin, desmin, epithelial membrane antigen, SOX10, and Cytokeratin (CK), but positive for CD34 (vascular+) (*Figure 3B*), CD31 (vascular+), S100 (scattered few+) (*Figure 3C*), Ki67 (30%+) (*Figure 3D*), vimentin (+++) (*Figure 3E*), CD68 (histiocyte+) (*Figure 3F*), CD163 (+++) (*Figure 3G*), and CK (−) (*Figure 3H*). MDM2 gene amplification was not detected by fluorescence *in situ* hybridization (FISH). Based on the immunohistochemical and morphological findings, the diagnosis of arterial intimal sarcoma was confirmed.

### Outcome and follow-up

Postoperatively, the oncologist recommended systemic cyclic chemotherapy. However, the patient opted to delay chemotherapy until full recovery from surgery due to his poor postoperative condition. At the time of this report’s submission, the patient is 2 years postoperatively and has not received chemotherapy. There are no indications of tumour recurrence or systemic metastasis, and the patient is in a generally stable condition.

## Discussion

Pulmonary artery intimal sarcoma is an exceptionally rare malignancy, with an estimated prevalence of 0.001%–0.03%.^1,4^ The mean age at diagnosis is 48 years (range: 13–86 years), and it is slightly more common in women.^3^ Tumours originating from the intima of elastic arteries, such as PAIS, typically grow intraluminally along the intimal layer of large vessels, including the pulmonary arteries and aorta. This growth pattern can lead to obstruction of major vessels or distal embolization.^5^ The diagnostic challenge associated with PAIS often stems from its frequent misdiagnosis as PE, which can delay effective management. Key clinical and imaging features may aid in distinguishing PAIS from PE. For instance, chronic progressive dyspnoea that is refractory to anticoagulation, as observed in our patient, contrasts sharply with the acute onset typically seen in PE. Furthermore, fixed irregular masses detected via echocardiography, along with luminal stenosis and heterogeneous contrast enhancement on CT (*Figure 1C* and *D*), strongly suggest the presence of sarcoma. In contrast, thrombi usually appear with varying degrees of mobility and are non-enhancing. The absence of thrombotic risk factors, such as immobility or hypercoagulability, should also heighten suspicion for malignancy. Pulmonary artery intimal sarcoma was first identified by Mandelstamm in 1923 during an autopsy,^5^ and only a few hundred cases have been reported globally. Surgical resection remains the primary treatment, although the roles of adjuvant radiotherapy and chemotherapy are uncertain. The prognosis for PAIS is poor, with a median survival of 1.5 months without surgical resection, extending to 36.5 months with complete resection and 11 months with incomplete resection.^6–9^

The diagnostic challenge in this case highlights the difficulty of identifying PAIS. The patient presented with worsening dyspnoea, a symptom associated with numerous conditions, prompting initial diagnoses of more common and potentially fatal conditions such as PE or heart failure secondary to myocardial infarction. Other differential diagnoses include pulmonary artery leiomyosarcoma,^10^ typically showing homogeneous enhancement. Takayasu arteritis,^11^ characterized by circumferential wall thickening, and metastatic disease, which is usually multifocal. For cases with equivocal findings, cardiac magnetic resonance (CMR) typically offers superior soft-tissue characterization of cardiac tumours, significantly aiding visualization of internal tumour architecture. Alternatively, positron emission tomography-computed tomography (PET-CT) should be considered for guiding biopsy if it demonstrates fluorodeoxyglucose (FDG) avidity [maximum standardized uptake value maximum (SUV_max_) > 5)] or persistent filling defects despite therapeutic anticoagulation. However, the tumour’s location in the main pulmonary artery near the pulmonary valve contributed to the detection of accelerated blood flow signals on echocardiography. Enhanced CT further delineated the tumour’s location and extent, ultimately leading to surgical intervention and pathological confirmation.

## Conclusion

This case underscores the importance of considering rare diseases such as PAIS in patients with unexplained dyspnoea, particularly when common diagnoses and treatments fail to resolve symptoms. Clinicians should maintain a broad differential diagnosis to avoid delays in identifying and managing uncommon but life-threatening conditions.

## Lead author biography


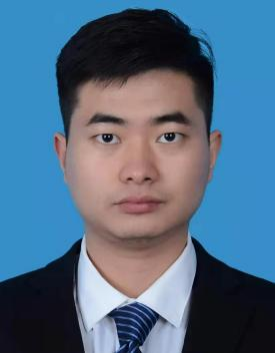


Lei Liu is an attending physician in the Department of Ultrasound Medicine at Nanjing Gulou Hospital. He has 8 years of clinical experience and has been practicing cardiac ultrasound diagnosis for more than 6 years. He has published several papers on cardiac Science Citation Index.

## Data Availability

The data underlying this article will be shared on reasonable request to the corresponding author.
